# Readiness of Pharmacists Based in Utah About Pain Management and Opioid Dispensing

**DOI:** 10.3390/pharmacy7010011

**Published:** 2019-01-15

**Authors:** Meghan M Balough, Stephen Nwankpa, Elizabeth J Unni

**Affiliations:** 1 Utah Department of Health, Salt Lake City, UT 84114, USA; mbalough@utah.gov; 2 Roseman University of Health Sciences College of Pharmacy, 10920 South River Front Parkway, South Jordan, UT 84095, USA; snwankpa@student.roseman.edu

**Keywords:** pharmacists, pain management, opioids, naloxone, pharmacist role

## Abstract

Prescription opioid use disorder is a growing epidemic and pharmacists as the dispensers of prescription drugs can play a crucial role in the management of the opioid crisis. However, few studies have examined pharmacists’ perceptions of their role in it. The objective of this study was to evaluate the perceptions of pharmacists in Utah regarding their role in the opioid epidemic. The study utilized a cross sectional online survey design to understand the pharmacist knowledge and beliefs regarding pain management, opioids, naloxone, and the various opioid risk identification tools. Frequencies, *t*-tests, and chi-squared were used to describe and analyze the data. A total of 239 surveys were qualified for analysis. Analysis showed that pharmacists have positive attitudes towards opioid crisis management; however, this positive attitude was higher among newer pharmacists. Though the pharmacists were knowledgeable with the opioid pharmacotherapy and prescribing guidelines, they demonstrated education needs for hands-on training when faced with a situation of prescription opioid use disorder in their practice. The use of risk identification tools was not prevalent. Results show lack of active participation by pharmacists in this major public health challenge, and the need for education in several aspects of opioid dispensing, naloxone use, and efficient use of risk identification tools.

## 1. Introduction

Opioids are a class of drugs that includes everything from legal prescription pain relievers such as oxycodone, hydrocodone, codeine, morphine, and fentanyl, to illicit drugs such as heroin [1]. The prescription opioids were promoted by pharmaceutical companies to treat moderate-to-severe pain or chronic pain from health conditions such as cancer with no potential risk [2]. Opioids were prescribed without any restriction on the quantity and indication until abuse potential of opioids leading to dependence and addiction became a high-priority concern globally [2]. Providers wrote nearly a quarter of a billion opioid prescriptions in 2012, enough for every American adult to have their bottle of thirty pills—with the number of prescriptions having a wide variation across states [3]. However, this reflected no change in the amount of pain reported by Americans, but a parallel increase in the opioid-related incidence of addiction and dependency [3]. Consequently, this has resulted in escalated use and abuse of opioids in the United States. In 2010, the Centers for Disease Control and Prevention (CDC) characterized the problem as a “growing, deadly epidemic” [3]. According to a recent White House report (2017), the opioid crisis (both prescription and illicit opioids) is considered a nationwide public health emergency. Nearly 175 Americans die every day from opioid overdose, which is almost seven individuals per hour, higher than the number of individuals dying from firearms and motor vehicle crashes [4]. “Opioid epidemic” is a term used by the U.S. Department of Health and Human Services to describe this health crisis arising from the misuse, abuse, and overdose of both prescription opioids such as oxycodone, hydrocodone, codeine, and morphine; and illicit opioids such as heroin and other synthetic opioids. Misuse of prescription opioids refer to taking opioids in a manner or dose other than prescribed for its intended purpose. Abuse of prescription opioids refer to taking someone else’s prescription, or taking the opioid to feel euphoria. 

Many national pharmacy organizations believe that pharmacists as drug experts have unique educational training, skills, and responsibilities to be actively involved in the fight against the opioid epidemic. For instance, American Pharmacists Association (APhA) strongly advocates for the recognition of pharmacists as healthcare providers who must exercise professional judgment in the assessment of a patient’s conditions to fulfill the corresponding responsibility of the use of controlled substances [5]. In other words, when opioids are prescribed not in accordance with the appropriate guidelines, the pharmacists can take steps to correct this prescription. They can also educate physicians on opioid prescribing guidelines and patients on the appropriate use of opioids. Recently, Walmart Pharmacy doubled its efforts associated with the Opioid Stewardship Initiative, intended to curb the spread of prescription opioid use disorder, prevent overdose, and reduce over-prescription from prescribers [6]. Though there has been a declining trend in overall opioid prescribing in the United States since 2012, the amount of opioids in morphine milligram equivalents prescribed per individual is still high [7]. 

In Utah, the opioid crisis has grown exponentially since the year 2000 [8]. The CDC ranks Utah as one of the highest high-dosage opioid prescribing states [9]. The majority (55%) of deaths from prescription pain medications in Utah involve oxycodone, and risk of death is significantly higher when methadone is involved [9]. The Utah Department of Health (UDOH) reports an increase in the rate of opioid prescription dispensed per 1000 population from 686.4 to 888.5 between 2002 and 2015. In 2015, the number of emergency department visits related to opioid overdose per 10,000 population was 13.3 among 45–54-year-olds, and 21.9 among 25–34-year-olds in Utah [8]. In 2016, the Opiate Overdose Response Act was passed in Utah that allows pharmacists to dispense naloxone (opioid reversal medication) as needed with a standing order or collaborative practice agreement. This came two years after the Good Samaritan Law (House Bill 11, Overdose Reporting Amendments), which enables bystanders to report an overdose without fear of criminal prosecution; and the Naloxone Access Law (House Bill 119, Opiate Overdose Emergency Treatment), which permits physicians to prescribe naloxone to third parties. The CDC published guidelines for prescribing opioids for chronic conditions in 2016 [8]. These guidelines were intended to promote thorough assessments for the need of prescription opioids, closely monitor the risks, and safely discontinue prescription opioids. In comparison to the World Health Organization’s treatment guidelines on pain, the CDC guidelines is more specific about opioid use in chronic pain. A major recommendation from the CDC guidelines is the use of state prescription drug monitoring programs such as the Controlled Substance Database (CSD) to review the patient’s history of controlled substance prescriptions to determine if the patient is at high risk for opioid overdose or prescription opioid use disorder. 

Many editorials and opinion articles have highlighted the significant role of the pharmacist in curbing the opioid epidemic; however, few studies have examined pharmacists’ perceptions of this specific role [10,11,12]. Thus, the overarching goal of this study was to understand how pharmacists in Utah perceive their specific role in curbing the effect of the opioid epidemic, specifically the over prescription of opioids. Primary prevention efforts can include collaborating with physicians, educating physicians, educating and counseling patients, and accepting the role of gatekeepers of opioid prescriptions. The specific objectives of the study were to evaluate pharmacist’s attitude towards their role in reducing the over-prescription of opioids, their knowledge about opioids and pain management, their awareness and familiarity with the various tools and resources that can be used to reduce the over-prescription of opioids, and their educational needs in reducing prescription opioid use disorder.

## 2. Materials and Methods

This study utilized a cross-sectional survey design using an online survey platform. The survey link was sent by email to all licensed pharmacists in Utah. The list of licensed pharmacists was obtained from the Utah Department of Occupational and Professional Licensing (DOPL). The survey had a total of 41 items and six sections that included: (1) attitudes—pharmacist attitudes regarding their role in reducing the opioid epidemic, collaborating with physicians and recommending changes to reduce the over-prescription of opioids, counseling patients on prescription opioid use disorder, use of naloxone, and their opinion on the use of Controlled Substance Database (CSD); (2) knowledge—pharmacist knowledge regarding pain management, prescribing guidelines on opioids, and use of the CSD; (3) awareness—pharmacist awareness of opioid prescribing guidelines, the Opiate Overdose Response Act, the CSD, and risk-identifying tools when dispensing opioids; (4) the various tools and resources used by pharmacists in reducing opioid addiction; (5) the educational needs of the pharmacists in reducing opioid addiction; and (6) other specific items such as whether the pharmacy stocks naloxone and have policies regarding opioid/naloxone dispensing. 

There were 13 attitude items on a scale of 1 (strongly disagree) to 5 (strongly agree). Three items were about their attitudes towards collaborating with physicians, four about their role in opioid crisis management, and six items on their attitudes towards dispensing and counseling patients on opioids and naloxone. A total attitude score (ranging from 13 to 65) was calculated by adding all 13 attitude items, in addition to separate scores for their attitudes towards physicians (ranging from 5 to 15), their role (ranging from 5 to 20), and patients (ranging from 5 to 30). Higher scores represent favorable attitudes. To determine pharmacists’ knowledge regarding the use of opioids for treatment of acute or chronic pain, six knowledge items were used and a total score was calculated by adding all six knowledge items. Higher scores represent better knowledge of proper use of opioids for pain management. Pharmacists’ awareness/familiarity with available resources that aim to lessen the over-prescription of opioids were measured using a 5-point Likert scale ranging from “not at all familiar” to “very familiar”. Pharmacists were also asked about the tools and resources they currently use such as the CSD or any other risk assessment. Pharmacist education and training needs were measured by using a “select all that apply” question with 15 varied educational goals. These included items such as naloxone dispensing, opioid prescribing guidelines, tapering patients off opioids, using the CSD, and consulting with physicians on pain management. 

The last section on demographics collected data on both the pharmacist’s personal demographics such as age, professional demographics such as education and credentials, and practice demographics such as naloxone stocking. Descriptive statistics such as frequencies and means were used to describe the central tendency of the data. *t*-tests were used to identify differences in the mean knowledge, attitudes, awareness, and utilization of tools based on the variables of gender, age, years of experience, credentials, primary practice site, primary place of practice, number of pharmacists, and number of prescriptions being filled. The gender variable was a dichotomous variable as male and female. Age group was classified as those 34 years of age or younger and 35 or older. Years of experience was classified as 5 years or younger versus all other. Similarly, primary place of practice and primary setting of practice were dichotomous variables (urban and rural/frontier, community/independent pharmacy and other, respectively). The credentials of pharmacists were dichotomized as PharmD versus other (BSPharm, residency or fellowship-trained, and board-certified). The pharmacy practice site was dichotomized as independent/community pharmacies versus other (ambulatory care clinic, hospital, long-term care, and academia). The number of pharmacists working in the pharmacy at the same time was classified as one versus two or more. The number of prescriptions dispensed was classified as less than 200 and 200 or more. Chi-squared was used to identify the differences in the proportions of respondents in each variable category regarding their knowledge, attitudes, awareness, and utilization of tools for the variables years of experience, primary place of practice, age, gender, and credentials. The analyses were carried out using SPSS version 23^®^ (IBM, Armonk, NY, USA) and SAS 9.4^®^ (SAS Institute, Cary, NC, USA).

## 3. Results

Data were collected from licensed pharmacists in Utah in July 2017. Of the 2460 survey invitations that were sent through email, 2302 emails were delivered. The 239 responses that were received were used in the analysis. Due to limitations of the DOPL dataset, such as outdated and/or inaccurate emails, 239 responses were considered a convenience sample in this analysis. The majority of respondents were males (66%), and more than half were aged 35 years or older (55%). Nearly 84% had PharmD as the educational qualification, and more than half of respondents (57.08%) had been in practice for five years or longer. When asked about workflow at their practice site, 44% reported having only one pharmacist at a time in the pharmacy, 34% reported two pharmacists, and 22% reported three or more pharmacists at any given time. The majority of the respondents reported practicing in an independent/community pharmacy setting (66.5%) and practicing in an urban area (86.63%). On a typical weekday, almost 60% of pharmacists reported filling more than 200 prescriptions, and on a typical weekend day, less than 20% filled more than 200 prescriptions. On questions regarding naloxone, 85.3% reported stocking naloxone in the pharmacy, and 77.3% reported having policies regarding opioid/naloxone dispensing. Table 1 describes the respondent demographics in detail. 

### 3.1. Pharmacist Attitudes Toward their Role in Reducing the Over-Prescription of Opioids

**Figure 1:**
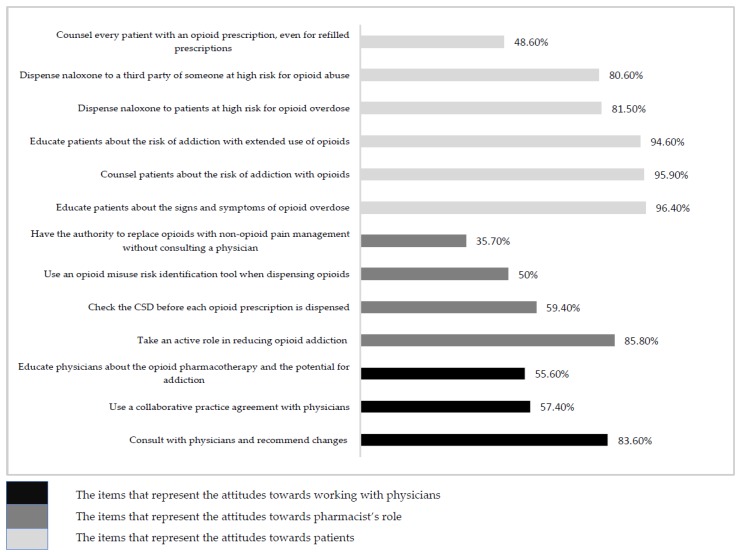
Pharmacist attitudes toward their role in reducing the over-prescription of opioids. Notes: CSD: Controlled Substance Database. The percentages represent the percentage of pharmacists who either agreed or strongly agreed on the various attitude items.

The mean total attitude score of the respondents were 50.71 (SD 7.96). Their attitude towards their role was 14.32 (SD 3.24), towards working with physicians in opioid crisis management was 11.22 (SD 2.6), and towards educating patients was 25.18 (SD 3.8). Although 85% of the respondents reported (agreed or strongly agreed) that pharmacists should take an active role in reducing opioid addiction, only one-third reported using an opioid misuse risk identification tool. Though almost 60% of the respondents agreed that they should check the CSD before dispensing any opioid prescription, 52% reported using the CSD with every new opioid prescription and less than 30% reported using it with every opioid refill prescription. While more than 80% of the respondents agreed that pharmacists should recommend changes to physicians when opioids are prescribed outside opioid prescribing guidelines, only 57% agreed that it is the pharmacist’s role to educate physicians about opioid therapy, and only 36% agreed that pharmacists should have the authority to replace opioids with non-opioid pain management. Though almost all pharmacists (96%) agreed that they should educate patients on the signs of opioid overdose and risk of opioid addiction, and approximately 80% agreed that pharmacists should be dispensing naloxone to high-risk patients and third parties like family and friends, only less than 50% agreed that they should be counseling every patient with an opioid prescription. Figure 1 demonstrates the attitudes of pharmacists about their role in opioid crisis management.

Newer pharmacists (those who have been practicing for less than five years) had better total attitude towards their role in opioid crisis management (52.37 vs. 49.48; *p*-value = 0.009), including their attitude about educating and counseling patients on opioids and naloxone dispensing (25.82 vs. 24.65; *p*-value = 0.024). Newer pharmacists were also more likely to agree that pharmacists can use collaborative practice agreements with physicians for pain management (67.0% vs. 50.4%, *p*-value = 0.01); that pharmacists should have the authority to replace opioids with non-opioid pain management after patient counseling without consulting a physician (43.6% vs. 30%, *p*-value = 0.04); and that pharmacists should use an opioid misuse risk identification tool when dispensing opioids (59.6% vs. 43%, *p*-value = 0.01). Those who practice at sites other than independent community pharmacies were more likely to agree that pharmacists should educate physicians about the pharmacotherapy of the different opioids available and the potential for addiction (67.6% vs. 48.9%, *p*-value = 0.01) and had better attitudes regarding their roles with physicians (73.2% vs. 57.6%, *p*-value = 0.03).

### 3.2. Pharmacist’s Knowledge about Opioids and Pain Management 

**Figure 2:**
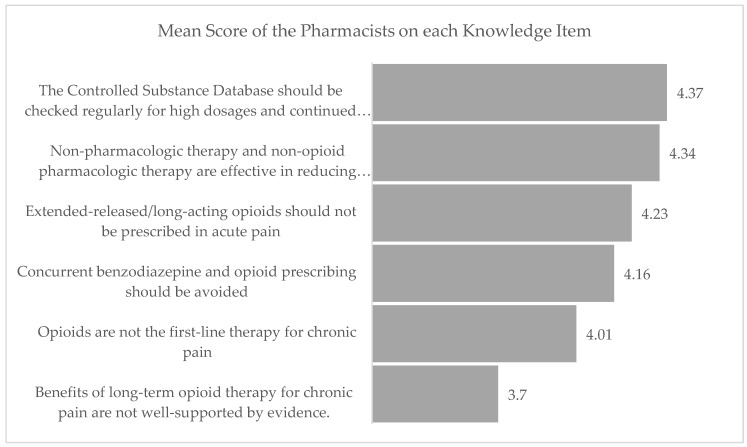
Mean score of the respondents on the knowledge items. Note: Each knowledge item was measured on a 5-point Likert scale with 1 representing “strongly disagree” and 5 being “strongly agree”. Higher scores indicate better knowledge on each item.

Of the possible 30 points that can be achieved on knowledge, the mean knowledge score was 24.83 (SD 3.123). A little over 70% of the respondents had a score equal to or greater than 80%. In general, the knowledge scores showed no difference based on demographics except for the area of practice. Pharmacists who practiced in in urban areas had significantly higher mean knowledge scores than those in rural areas (mean 25.2 vs. 23.5, *p*-value = 0.02). Figure 2 describes the items and the mean score on each items. 

### 3.3. Pharmacist Awareness and Familiarity with Various Tools and Resources That Can be Used to Reduce the Over-Prescription of Opioids

**Figure 3:**
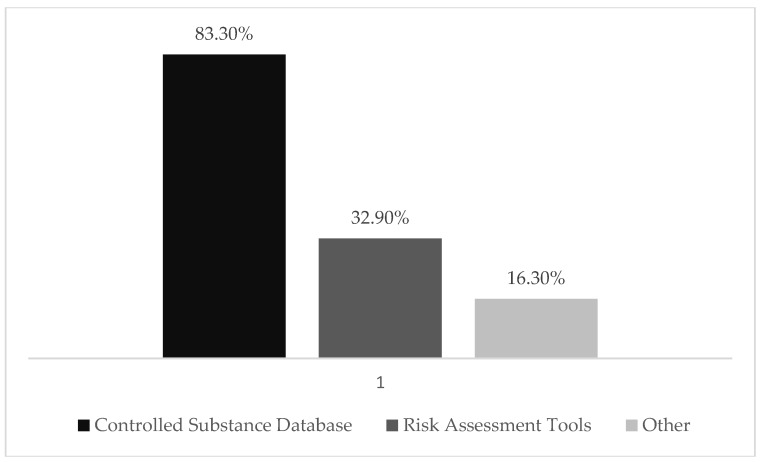
Percentage of pharmacists who reported the use of various resources to reduce the over-prescription of opioids. Note: “Other” was a choice given to respondents, followed by an open-ended question that said, “please explain”.

When asked about familiarity with the CSD, almost 90% were familiar with the CSD. However, when asked the frequency of using the CSD as a tool/resource, 83.3% reported using it, and a little more than half (52.1%) reported checking the CSD with every new opioid prescription (Figure 3).

Less than half (42.4%) reported being moderately or very familiar with CDC prescribing guidelines and only about a third (33.1%) reported being moderately or very familiar with the Opiate Overdose Response Act. Pharmacists with credentials other than PharmD were less familiar with CDC guidelines (39.6% vs. 60.0%; *p*-value = 0.03) and Opiate Overdose Response Act (29.0% vs. 51.4%; *p*-value = 0.001). When asked to report the “other” tools used for risk assessment, it was mainly patient consultation and checking the patient profile.

### 3.4. Education and Training Needs Identified by Pharmacists

**Figure 4:**
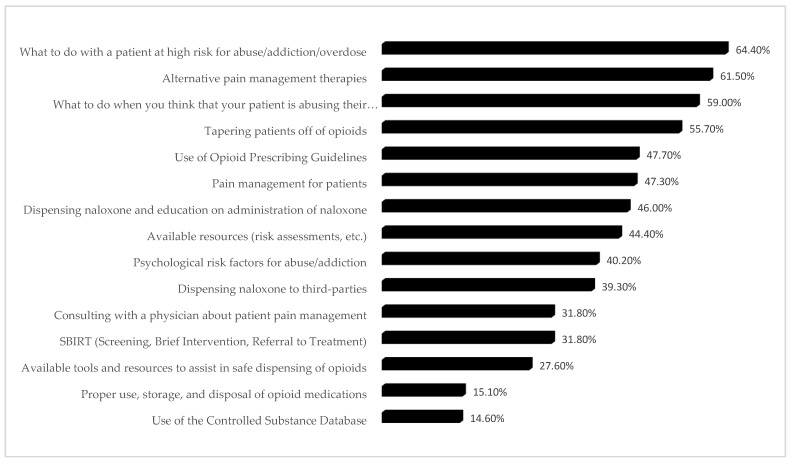
Priority education needs for pharmacists.

Only approximately 50% of respondents provided feedback regarding educational priorities. More than 50% of the respondents selected the following: (1) what to do when the patient may be abusing their medication, (2) alternative pain management, (3) how to manage patients at high risk for addiction, and (4) tapering patients off opioids as their education priority. With more than 80% of pharmacists using CSD, they did not believe they need further training on it as well as the use, storage, and disposal of opioids. While 44% wanted further education on risk assessment resources, 32% indicated the need for further education and training on the use of Screening, Brief Intervention, Referral to Treatment (SBIRT). The other educational priority was training on naloxone, both administration (44.4%) and dispensing (39.3%). Figure 4 describes in detail the educational priorities selected by the respondents.

## 4. Discussion

Previous literature has highlighted the significant role that can be taken by pharmacists in reducing prescription opioid use disorder. Some recent studies examined the willingness and readiness of pharmacists in dispensing naloxone [13,14,15,16]. However, hardly any studies have examined pharmacists’ perceptions of their roles in opioid crisis management, which includes their knowledge about pain management, alternative pain management, naloxone dispensing, collaborating with physicians on opioid management, educating physicians about opioids and naloxone, and counseling patients on opioids and naloxone. This study measured pharmacists’ perceptions and knowledge towards their role in opioid management and how it could change the course of opioid crisis. The overall study results indicate that pharmacists have positive attitudes towards opioid management; however, this positive attitude was more for newer pharmacists, those who have been practicing for less than five years. Though there was no difference in overall knowledge, the newer pharmacists were more inclined towards educating and counseling patients on opioids and naloxone dispensing, their role with physicians such as collaborative practice agreements for pain management, and their own roles including the authority to replace opioids with non-opioid pain management without consulting a physician. Potentially, the increased awareness about opioid crisis in the recent years may have prepared the newer pharmacists for their role in this crisis management. When asked about working with physicians in opioid crisis management, pharmacists who were practicing at sites other than community/independent pharmacies such as ambulatory care clinics, hospitals, and long-term care facilities had better attitudes. However, interestingly, the study was not able to find any difference in the attitude or knowledge based on the number of prescriptions or the number of pharmacists working in the pharmacy, which can be proxy variables for the busyness of the pharmacy or time constraint of the pharmacists. One potential explanation for this can be that pharmacies other than community/independent pharmacies may have a better work flow that allows them to spend increased time with physicians. An earlier study by Bradshaw and Doucette on physician’s attitudes towards community pharmacists in Utah demonstrates the importance of working conditions that allow pharmacist/patient interaction as mechanisms that would assist physicians in coordinating pharmacotherapy and providing patients with medication information [17]. Additionally, unlike pharmacists who work in hospital settings, community pharmacists do not have dedicated time to update their knowledge about the various new developments in pharmacotherapy. Thus, improved work flow and dedicated time for knowledge gain may support for community pharmacists to play a major role in public health issues such as opioid crisis management. It is also possible that the work ergonomics in community pharmacies (such as drive-through pharmacies), lack of mandatory break time, or the constant flow of patients may be contributing towards the less favorable attitudes of community pharmacists towards their role in opioid crisis.

Another interesting observation from the study was the training needs requested by the respondents. Though more than 70% of the respondents scored more than 80% on the knowledge questions, more than 50% of pharmacists identified “what to do when the patient may be abusing their medication”, “alternative pain management”, “how to manage patients at high risk for addiction”, and “tapering patients off opioids” as their educational priorities. Though the pharmacists are knowledgeable with the opioid pharmacotherapy and prescribing guidelines, they may be needing hands on training when faced with a situation of prescription opioid use disorder in their everyday practice. The recent publication by the College of Psychiatrists and Neurologic Pharmacists on Opioid Use Disorders, “Interventions for Community Pharmacists”, can be an appropriate guideline to educate community pharmacists on interventions to provide safe and appropriate access to opioids while protecting the public from the hazards of prescription opioid use disorder [18]. Additionally, the three drugs approved by the U.S. Food and Drug Administration for the treatment of opioid use disorder, namely, buprenorphine, methadone, and naltrexone, represent be another topic for educating pharmacists in this public health challenge.

Lack of integrated continuous education for community pharmacists may also account for the limited use of the various risk assessment tools to ensure comprehensive control and monitoring of opioid prescriptions. The study results showed that most pharmacists were aware of the CSD and reported using it, there were little or no references to other resources such as SBIRT. Also, only less than half of the pharmacists reported familiarity with CDC guidelines for pain management and only a third reported familiarity with the Opiate Overdose Response Act. Additionally, it was observed that only half of the pharmacists were utilizing the CSD for every new opioid prescription (even though Utah requires pharmacists to check this resource for every new patient). Perhaps this may be happening due to lack of time. This might also be the reason that only 60% of the pharmacists either agreed or strongly agreed with the statement that “Pharmacists should check CSD with every opioid prescription”. Though 80% of respondents felt comfortable dispensing naloxone to high-risk patients and their families, 46% of respondents identified “dispensing naloxone and education administration” as an educational need. These numbers show the gap between attitude and knowledge of pharmacists with regard to naloxone dispensing. Compared to other studies that reported the comfort level of dispensing naloxone, this study only measured their attitude towards naloxone dispensing. That may be the reason why the results from this study are higher than those reported by Thornton et al. where only 20% of community pharmacists in West Virginia felt comfortable dispensing naloxone without a prescription and 37% in Kentucky [19].

The study is not without limitations. A major limitation is the small convenience sample which was used for analysis that can influence the generalizability of the study. The lack of an accurate database of registered pharmacists makes it difficult to access the pharmacists for survey designed study. The survey used in the study is not a validated scale. The scale was developed by the Utah Department of Health with input from pharmacists. As with any self-reported survey, skewed responses due to social desirability bias is a potential limitation. Finally, this study only examined the readiness of pharmacists in reducing the over-prescription of opioids and has not examined or discussed the various other reasons that are causing this epidemic and/or the roles of other healthcare providers in this public health challenge.

## 5. Conclusions

While there is a push to increase the pharmacist’s role in reducing prescription opioid use disorder, this study demonstrates the varying attitudes of pharmacists towards this role. While the majority of pharmacists think they should educate patients on opioid overdose and risk of addiction, and that they should play an active role in reducing opioid addiction or recommend changes to physicians when opioids are prescribed differently to CDC guidelines, only half of them think it is their role to educate physicians about opioid pharmacotherapy. Furthermore, only a third think they should have the authority to replace opioids with non-opioid pain management. The perception of a limited role among pharmacists may be stemming from several factors discussed earlier, including lack of time due to workload, lack of hands-on training (especially with prescription opioid use disorder), and lack of familiarity with risk assessment tools. This may also be due to the fact that pharmacists are unsure of their role in this public health crisis and the potential positive changes that can come from taking a more active role with other healthcare providers. The results from this study can be used by the Utah Pharmacists Association or the colleges of pharmacy in Utah to provide further education and empower pharmacists in recognizing and acting on their role in this crisis. 

## Figures and Tables

**Table 1:** Respondent demographics (the total number of respondents was 239).

	*N*	%
**Experience as a Pharmacist**		
Less than 5 years	103	42.9
5 to 10 years	69	28.7
11 to 15 years	24	10
More than 15 years	44	18.4
**Pharmacist Credentials**		
PharmD	177	83.5
BSPharm	32	15.0
Other (Residency/fellowship, board-certified)	3	1.5
**Primary Place of Practice**		
Urban	149	86.6
Rural/frontier	23	13.4
Pharmacy practice site		
Community/independent pharmacy	141	66.5
Ambulatory care clinic	13	6.3
Hospital	40	18.9
Long-term care	13	6.4
Academia	4	1.9
**Age Group**		
25 to 34	93	44.7
35 to 44	75	36.1
45 to 54	22	10.5
55 to older	18	8.7
**Gender**		
Male	137	65.9
Female	71	34.1

## References

[1] National Institute on Drug Abuse (2018). Opioids. https://www.drugabuse.gov/drugs-abuse/opioids.

[2] US Food and Drug Administration Timeline of Selected FDA Activities and Significant Events Addressing Opioid Misuse and Abuse. https://www.fda.gov/downloads/Drugs/DrugSafety/InformationbyDrugClass/UCM566985.pdf.

[3] Baldwin G. Overview of the Public Heakth Burden of Prescription Drug and Heroin Overdoses. https://www.fda.gov/downloads/drugs/newsevents/ucm454826.pdf.

[4] The President’s Commission on Combating Drug Addiction and the Opioid Crisis. https://www.whitehouse.gov/sites/whitehouse.gov/files/images/Final_Report_Draft_11-1-2017.pdf.

[5] (2004). American Pharmacists Association: American Pharmacists Association APhA Policy Manual. http://www.pharmacist.com/policy-manual?key=opioid.

[6] Walmart Pharmacy Opioid Stewardship Initiative: Guideline for Dispensing Opioid 2018. https://corporate.walmart.com/media-library/document/opioid-fact-sheet/_proxyDocument?id=00000163-3abc-ded8-ab7f-3ffe314e0000.

[7] Centers for Disease Control and Prevention, Prescription Opioid Data. https://www.cdc.gov/drugoverdose/data/prescribing.html.

[8] Centers for Disease Control and Prevention Guideline for Prescribing Opioids for Chronic Pain. https://www.cdc.gov/drugoverdose/pdf/guidelines_factsheet-a.pdf.

[9] Utah Department of Health Utah Violent Death Reporting System (2017). Opioid-Related Emergency Department Visits Per 10,000 Population by Age Group, Utah. Utah. https://ibis.health.utah.gov/indicator/view/PoiDth.0_24.html.

[10] Thomas E., Meinghan B.M. (2016). Pharmacists are critical to curbing the opioid crisis. Pharm. Today..

[11] American Society of Health System Pharmacists (2016). ASHP Statement on the Pharmacist’s Role in Substance Abuse Prevention, Education, and Assistance. Am. J. Health-Syst. Pharm..

[12] Matthew P., Shearer M.A. (2017). Serving the Greater Good: Public Health & Community Pharmacy Partnerships. Johns Hopkins Center for Health Security. Johns Hopkins Bloomberg School of Public Health. http://www.centerforhealthsecurity.org/our-work/pubs_archive/pubs-pdfs/2017/public-health-and-community-pharmacy-partnerships-report.pdf.

[13] Shannon E., Rudolph A.R. (2018). Identifying barriers to dispensing naloxone: A survey of community pharmacists in North Carolina. J. Am. Pharm. Assoc..

[14] Bailey A.M., Daniel P.W. (2018). Naloxone for Opioid Overdose Prevention:Pharmacists’ Role in Community-Based Practice Settings. Ann. Pharmacother..

[15] Erin L., Thompson P.P. (2018). Dispensing Naloxone Without a Prescription: Survey Evaluation of Ohio Pharmacists. J. Pharm. Pract..

[16] Meyerson B.E., Agley J.D., Davis A., Jayawardene W., Hoss A., Shannon D.J., Ryder P.T., Ritchie K., Gassman R. (2006). Predicting pharmacy naloxone stocking and dispensing following a statewide standing order, Indian 2016. Drug Alcohol. Depend..

[17] Bradshaw J.S., Doucette W. (1996). Community Pharmacists as Patient Advocates: Physician Attitudes. J. Am. Pharm. Assoc..

[18] The College of Psychiatric and Neurologic Pharmacists (CPNP) (2016). Opioid Use Disorders: Intervention for Community Pharmacist. https://cpnp.org/ed/presentation/2016/opioid-use-disorders-interventions-community-pharmacists?view=link-0-1471880668.

[19] Patricia R., Freeman A.G. (2016). Pharmacists’role in opioid overdose: Kentucky pharmacists’ willingness to participate in naloxone dispensing. J. Am. Pharm. Assoc..

